# Avon Longitudinal Study of Parents and Children (ALSPAC) summary education attainment values in the offspring generation

**DOI:** 10.12688/wellcomeopenres.24082.1

**Published:** 2026-01-26

**Authors:** Eleanor I. Walsh, Mark Mumme, Kate Northstone

**Affiliations:** 1 Population Health Sciences, University of Bristol Medical School, Bristol, England, BS10 5FN, UK

**Keywords:** ALSPAC, cohort study, education, attainment, record linkage

## Abstract

This data note describes how summary education attainment scores were generated for the index participants of the Avon Longitudinal Study of Parents and Children (ALSPAC) from the original datasets provided by the Local Education Authorities and the Department for Education. Detailed education data can be sensitive and there is a risk of re-identification, so access is restricted through a secure Trusted Research Environment (TRE). The high level of security does impose some restrictions on ease of access and there are financial and resource costs to the researchers and the hosts.

There is a demand for minimal educational attainment variables to be made available, often as co-variates in broader analyses (as opposed to being the primary exposure or outcome of interest). Summary educational attainment measures are considered by the Department for Education (DfE) to be of low sensitivity and identifiability risk. A summary variable of attainment for Early Years and each Key Stage 1–5 has been made available. Final sample sizes for each of these scores is dependent on external factors including consent status, which can change regularly.

## Introduction

Since 1998, the Avon Longitudinal Study of Parents and Children (ALSPAC) has linked to educational data made available from regional and national records for the offspring generation of the study (known as G1). Educational attainment is a specific and versatile indicator that has been used extensively alongside the vast and growing catalogue of other health and lifestyle information provided by the children and their families taking part in the study ^1– 4^.

To protect participant identity and confidentiality, a variety of data from regional and national educational records can be accessed and linked to the ALSPAC cohort via a secure TRE. However, educational attainment scores are considered to be of low sensitivity and identifiability risk by the Department of Education (DfE) ^5^. They can therefore be made accessible outside a TRE, when appropriately derived, and subject to existing protocols required to access ALSPAC data for research purposes (e.g. proposal approval, data sharing & access agreement, consent & disclosure checks, publication agreements; see Data Availability).

### Educational system in the UK

In the UK, children start full time education in the academic year that they turn 5 years of age ^6^. In practice, children usually start school in the September following their fourth birthday and move up a school year each September. A child’s school journey is divided into a number of “key stages” (KS; See Table 1 below for details ^7^). “Early Years” (EY), KS1 and 2 make up primary education (up to age 11) and KS3 and 4 make up the compulsory component of secondary education (ages 11–16 years). KS5 (ages 16–18 years) did not become part of compulsory education and training until 2013; this usually involves Further Education (FE) vocational or training qualifications.

**Table 1.** Structure of UK schools and testing procedures.

Class	Age	Key Stage	Tests
-	3 - 4	Early Years	Local entry assessments
Reception	4 - 5		
Year 1	5 – 6	1	National tests and tasks in English & Maths
Year 2	6 – 7		
Year 3	7 – 8	2	National tests and tasks in English, Maths & Science.
Year 4	8 – 9		
Year 5	9 – 10		
Year 6	10 – 11		
Year 7	11 – 12	3	National tests and tasks in English, Maths & Science.
Year 8	12 – 13		
Year 9	13 – 14		
Year 10	14 – 15	4	Some Children take GCSEs *, GNVQs **, etc. Most Children take GCSEs *, GNVQs **, etc.
Year 11	15 – 16		
Year 12	16 – 17	5	GCE *** AS Levels GCE *** A Levels and other level 3 qualifications
Year 13	17 - 18		

* GCSE – General Certificate of Secondary Education ** GNVQ – General National Vocational Qualification *** GCE – General Certificate of Education

At the end of each KS, academic performance is assessed through compulsory national tests designed to match the national curriculum.

Here we describe the derivation of overall scores for educational attainment in EY and at each KS 1–5 (ages 3–18 years old) that aim to be non-disclosive yet maintain sufficient granularity for statistical utility, that cannot be reverse engineered to the original data. These cannot be compared to national standards of attainment (i.e. they have cohort specific distributions).

## Data linkage of ALSPAC to education records

### ALSPAC recruitment and consent

ALSPAC recruited pregnant women with an expected due date between April 1991 and December 1992, living in a defined area in and around the city of Bristol, UK ^8^. Initially, 14,541 pregnancies were enrolled resulting in 13,988 study children alive at one year of age. An additional 718 children, who met the original study eligibility criteria, but whose mothers had not joined the study during pregnancy, were recruited by age 18 years. Full details on ALSPAC are given in the cohort profiles ^8, 9^ and the study website contains details of all the data available through a fully searchable data dictionary and variable search tool ^10^.

The ALSPAC parents were first asked for permission to link to the education records of their children when attending a clinic at the age of 7, which ran between September 1998 and September 2000. In line with the Data Protection Act at the time, education data was not requested for children whose parents objected at the clinic - or for whom permission was subsequently withdrawn.

When the study children reached legal adulthood (age 18 years), they were sent 'fair processing' materials describing ALSPAC’s intention to link to their routine health and administrative data from national databases, including education, and gave them a clear means to consent or object via a written form. Participants were informed that ALSPAC would attempt to link to their records unless they opted out. Data were not extracted for participants who objected, or who were not sent fair processing materials. Participants were asked to consider allowing data linkage to health, education, crime and environmental data. The education was split into compulsory schooling (EY & KS 1–4), further education (KS5) and higher education, and the participants could allow, or opt out of, any or all of these three categories.

At the time of writing, 12,839 participants (of the 13,238 consent forms returned) allowed their education records to be accessed by ALSPAC, 399 had dissented. Note that these consent figures represent a point in time; consent is ongoing, and numbers have changed over time. The available data set is only of those who explicitly consented and those who chose not to opt-out.

### Education data linkage

**Figure 1.**
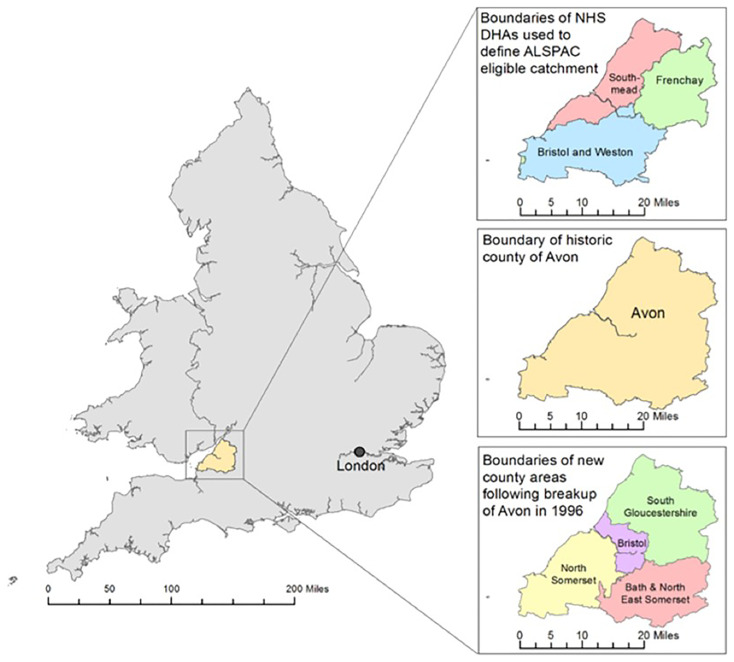
The ALSPAC Eligible Study Area within the UK: illustrating the NHS District Health Authorities (DHAs) used to define: the ALSPAC catchment area; the historical county of Avon; and the four authorities formed following the breakup of Avon. Contains Ordnance Survey, Office of National Statistics and National Records Scotland data © Crown Copyright/database right 2014.

On approval from the four Local Education Authorities (LEA) that covered the former Avon area (Bristol, South Gloucestershire, Bath & Northeast Somerset and North Somerset; Figure 1), ALSPAC’s data linkage team used deterministic linkage methodology. The School Entry Assessment and KS 1 records were linked to ALSPAC using names and dates of birth.

The National Pupil Database (NPD) was introduced by the DfE (or the Department for Children, Schools and Families (DCSF) as it was until 2010) in 2002. The NPD is a longitudinal database of pupils who attend state-maintained schools and colleges and includes records going back to academic year 1995/1996. The NPD holds detailed information about children’s education at different stages, exam and test results, absence and exclusion data, as well as pupil characteristic data such as ethnicity, language, free school meals entitlement, and details of any Special Educational Needs (SEN). The DfE introduced the Unique Pupil Number (UPN), which later became the Pupil Matching Reference–anonymous (PMRa), in 1999 to facilitate the collection of data across the school system. This was used to link participants’ identifiers to a systematically compiled pupil register.

In 2002, ALSPAC obtained linked NPD records for the ALSPAC participants. To satisfy the confidentiality requirements of both ALSPAC and the DfE, data linkage was carried out by an independent charity, The Fischer Family Trust (FFT), acting as a trusted third party. The DfE supplied the FFT with names, dates of birth and current postcodes of all the individuals in the NPD, and ALSPAC supplied similar details for all members of the eligible cohort. The FFT then linked ALSPAC identifiers to the DfE’s UPN by matching on the available data separately for each of the datasets held and maintained by the DfE. Some quality checks were required in a small number of cases where a single ALSPAC child was linked to different pupils in different years/datasets. A small number of bad matches were also identified by investigating cases for whom the reported national curriculum year was 2 or more years out from the expected national curriculum year.

The extract of the NPD data provided to ALSPAC contained datasets for schools in England from the academic years 1997/1998 to 2011/2012. Data has been available for research from 2004 for Entry Assessments and KS1, 2008 for KS2 and KS3, 2010 for KS4 and 2014 for KS5. The schools in this dataset were those recorded as the school at which the participants completed their KS assessments, KS 1 to 5, and were drawn from the DfE’s KS attainment datasets. The number of ALSPAC participants who were identified and matched by the DfE at each KS are presented in Table 2.

**Table 2.** The number of ALSPAC participants who were identified and matched by the DfE at each Key Stage.

Key Stage	Number
EY	13,301
KS1	11,279
KS2	12,233
KS3	10,851
KS4	11,764
KS5	9,449

The expected progress, according to date of birth, of each subdivision of the cohort through compulsory schooling and then followed by the KS5 route is shown in Table 3. Note that the oldest ALSPAC children entered reception year in autumn 1995 and the youngest took their KS5 assessments in summer 2011.

**Table 3.** Coding of academic years on ALSPAC data files and expected progress of the ALSPAC cohort according to their dates of birth. Expected position of ALSPAC children born between.

Academic year	Code	April 1991 & August 1991	September 1991 & August 1992	September 1992 & January 1993
1995 / 1996	1	Reception	-	-
1996 / 1997	2	Year 1	Reception	-
1997 / 1998	3	Year 2	Year 1	Reception
1998 / 1999	4	Year 3	Year 2	Year 1
1999 / 2000	5	Year 4	Year 3	Year 2
2000 / 2001	6	Year 5	Year 4	Year 3
2001 / 2002	7	Year 6	Year 5	Year 4
2002 / 2003	8	Year 7	Year 6	Year 5
2003 / 2004	9	Year 8	Year 7	Year 6
2004 / 2005	10	Year 9	Year 8	Year 7
2005 / 2006	11	Year 10	Year 9	Year 8
2006 / 2007	12	Year 11	Year 10	Year 9
2007 / 2008	13	Year 12	Year 11	Year 10
2008 / 2009	14	Year 13	Year 12	Year 11
2009 / 2010	15	-	Year 13	Year 12
2010 / 2011	16	-	-	Year 13

The national tests, assessing progress at the end of each KS, are detailed in Table 1. Local assessments were used at school entry, but these were the same across the ALSPAC geographical footprint.

## Summary variables

A decile score (a decile is a quantitative method of splitting up a set of ranked data into 10 equally large subsections) has been generated for EY and for each KS 1 to 5. The source and range of these scores vary for each KS and are described below.

It is important to note that these summary attainment scores for each participant show only the relative attainment within the ALSPAC cohort and therefore only facilitate comparisons through relative rankings within the cohort itself. They cannot be compared with national averages or maximums. Decile 1 is the group with the lowest valid scores and decile 10 is the group with the highest valid scores. Only participants with a valid score in each dataset are included. Participants might be excluded from a KS, i.e. not have a valid score, if they were not matched in that KS dataset, or were absent, ineligible, working below the level of the test, unable to access the test, had left, or their data was missing or null in the DfE dataset.

### Early years

Entry Assessment observes eight areas of experience. Four areas form the required element (language, reading, writing and mathematics) and the other four are voluntary (social skills, problem solving, large motor skills and small motor skills). Less than half the participants completed the voluntary elements, so only the required elements are included. Each area was scored by DfE from 2 to 7, these were re-coded by ALSPAC as 0 to 5 to create the EY summary score.

***EY score variable [variable label: clon455].*** The total score (i.e. from minimum 0 to maximum 20) from the four required elements was calculated for each of the 8,788 participants with valid scores and then grouped into deciles ( Table 4). 

**Table 4.** Number of participants in each decile ranking at each Key Stage of education.

	Early Years		Key Stage 1		Key Stage 2		Key Stage 3		Key Stage 4		Key Stage 5	
Decile ranking within ALSPAC of education attainment	[clon455]		[clon456]		[clon457]		[clon458]		[clon459]		[clon460]	
	(n)	(%)	(n)	(%)	(n)	(%)	(n)	(%)	(n)	(%)	(n)	(%)
**Not in dataset**	3,815	*22.8*	3,815	*22.8*	1,815	*10.8*	1,815	*10.8*	1,887	*11.3*	7,309	*43.6*
**Trip/quad**	6	*0.0*	10	*0.1*	6	*0.0*	6	*0.0*	6	*0.0*	6	*0.0*
**No data**	4,142	*24.7*	2,493	*14.9*	3,759	*22.4*	5,071	*30.3*	3,992	*23.8*		
**>1 Component Missing**	7	*0.0*	38	*0.2*	133	*0.8*	105	*0.6*			920	*5.5*
***sub-total (not in decile)***	*7,970*	*47.6*	*6,356*	*37.9*	*5,713*	*34.1*	*6,997*	*41.8*	*5,885*	*35.1*	*8,235*	*49.1*
**1 ^st^ (lowest)**	1,380	*8.2*	1,290	*7.7*	1,135	*6.8*	992	*5.9*	1,093	*6.5*	852	*5.1*
**2 ^nd^**	757	*4.5*	1,207	*7.2*	1,077	*6.4*	993	*5.9*	1,117	*6.7*	854	*5.1*
**3 ^rd^**	916	*5.5*	841	*5.0*	1,113	*6.6*	968	*5.8*	1,125	*6.7*	853	*5.1*
**4 ^th^**	1,012	*6.0*	944	*5.6*	1,134	*6.8*	997	*6.0*	1,223	*7.3*	888	*5.3*
**5 ^th^**	1,006	*6.0*	966	*5.8*	1,106	*6.6*	968	*5.8*	887	*5.3*	820	*4.9*
**6 ^th^**	944	*5.6*	1,791	*10.7*	1,088	*6.5*	1,001	*6.0*	1,127	*6.7*	936	*5.6*
**7 ^th^**	826	*4.9*	846	*5.1*	1,100	*6.6*	941	*5.6*	1,054	*6.3*	785	*4.7*
**8 ^th^**	690	*4.1*	980	*5.9*	1,109	*6.6*	981	*5.9*	1,122	*6.7*	853	*5.1*
**9 ^th^**	591	*3.5*	937	*5.6*	1,116	*6.7*	977	*5.8*	1,159	*6.9*	1,375	*8.2*
**10 ^th^ (highest)**	666	*4.0*	600	*3.6*	1,067	*6.4*	943	*5.6*	966	*5.8*	307	*1.8*
***sub-total (in deciles)***	*8,788*		*10,402*		*11,045*		*9,761*		*10,873*		*8,523*	

### Key stage 1

A total score had been calculated for KS1 ^11^ which summed the derived scores for Reading, Writing and Mathematics standardised tests (teaching assessments and spelling tests were not included) for n = 10,402 participants. These ranged from 0 to 5 for each element (maximum of 15). A prorated total score (scaled by a factor of 3/3 – n of missing scores) has been created, which includes pupils with no more than one missing result.

***KS1 score variable [variable label clon456].*** The summary score (maximum prorated summary score possible is 15, i.e. Reading max 5 + Writing max 5 + Maths max 5) was calculated for each participant with valid scores and then grouped into deciles ( Table 4).

### Key stages 2 and 3

KS2 ^11^ and KS3 ^11^ were broadly the same; both including English, Mathematics and Science, although the components and marking schemes are different. These are the first KS where pupils are also tiered for expected attainment.

Total score variables were available for English, Maths and Science which were summed to create a total score. KS2 and KS3 decile scores were obtained for n = 11,045 and 9,761 participants respectively. A prorated total score (scaled by a factor of 3/3 – n of missing scores) has been created, which includes pupils with no more than one missing result.

***KS2 score variable [variable label: clon457].*** The total score (the maximum score possible was 280, i.e. English max 100 + Maths max 100 + Science max 80) was calculated for each participant with valid scores and then grouped into deciles ( Table 4).

***KS3 score variable [clon458].*** The total score (the maximum score possible was 430, i.e. English max 100 + Maths max 150 + Science max 180) was calculated for each participant with valid scores and then grouped into deciles ( Table 4).

### Key stage 4

KS4 ^11^ includes both GCSEs & GNVQ equivalents. These involve a broader curriculum with multiple subjects and assessment components, some are compulsory, and others are optional.

We were provided with a score for overall KS4 attainment for GCSEs and equivalents for 10,873 participants. This was capped to control for very high achieving students who are entered for extra assessments (there are n = 46 total scores above the cap in the ALSPAC dataset which have been capped at 464, the maximum).

The capped scores equate to 8 A* GCSEs (8 A* = 58 x 8 = 464), but *“In practice 0.05% of the sample have scores slightly above 464 which may result from students taking qualifications which are more challenging than GCSEs (e.g. AS levels), or from small data errors.” ^12^*

***KS4 score variable [clon459].*** The total capped score was calculated for each participant with a valid score and then grouped into deciles. A prorated score was not required for the KS4 deciles ( Table 4).

### Key stage 5

KS5 ^11^ includes A-levels and equivalents which involve a broad curriculum with multiple subjects and assessment components, and there is no minimum requirement. KS5 is the last stage of secondary education in the UK. It is the two years of education for students in England, Wales and Northern Ireland, aged 16–18 and is usually the stage that allows students to apply to a university.

The NPD includes a score for overall KS5 attainment for A-levels and equivalents. There is no cap for the total KS5 score, and the ALSPAC participant’s scores range from 0 to over 1,800. The number of subjects (called entries by the DfE) varied, and many subjects counted for less than a full entry. Students generally take 3, or sometimes 4, entries at KS5, but there is no strict maximum or minimum. The ALSPAC participants in the KS5 dataset took from 1.0 to 8.3 entries.

To obtain a KS5 score which enables comparison across the cohort it was determined to generate a score as an average score per entry (i.e. subject). This approach was chosen so students who scored highly in a small number of entries would not be confused with students who scored lower but across a larger number of entries. The average score per entry was calculated from the total GCE A Level and equivalent points score (as designated by the Qualifications and Curriculum Authority (QCA), which has now been replaced by Office of Qualifications and Examinations Regulation (Ofqual) and Qualifications and Curriculum Development Agency (QCDA)) and divided by the total number of GCE A Level and A Level equivalent entries.

***KS5 score variable [variable label: clon460].*** The average score per entry was calculated for n = 8,523 participants with a valid score and then grouped into deciles ( Table 4).

## Ethics and consent

Ethical approval for the study was obtained from the ALSPAC Ethics and Law Committee (ALEC; IRB00003312) in June 2010 and the Local Research Ethics Committees (NHS Haydock REC: 10/H1010/70). The education data was provided by the DfE following their internal decision-making process, under Data Request Number DR120430.04 (dated 30/04/2012), which states that ‘ALSPAC operates as a resource for the entire research community’ and ‘The [education] data are pseudonymised and then used by researchers and research projects approved by the ALSPAC executive.’ Study involvement of the index participants (the children born in 1991–92) was based on parental approval until the children reached adulthood. Informed consent for the use of data collected via questionnaires and clinics was obtained from participants following the recommendations of ALEC at the time. When participants reached age 18 years they were sent ‘fair processing’ materials that invited them to continue to take part in ALSPAC and informed them about ALSPAC’s intention to link to their routine health and administrative records, including any education records, and gave a clear means to opt out. Where practicable (e.g. when attending a study assessment visit), participants were also able to explicitly consent.

## Data Availability

If you require further information about education attainment scores, please contact data@bristol.ac.uk. ALSPAC data access is through a system of managed open access. Please see the ALSPAC data management plan which describes the policy regarding data sharing ( http://www.bristol.ac.uk/alspac/researchers/data-access/documents/alspac-data-management-plan.pdf), which is by a system of managed open access. The steps below highlight how to apply for access to the data included in this study and all other ALSPAC data: i. Please read the ALSPAC access policy which describes the process of accessing the data and samples in detail, and outlines the costs associated with doing so. ii. You may also find it useful to browse our fully searchable research proposals database, which lists all research projects that have been approved since April 2011. iii. Please submit your research proposal for consideration by the ALSPAC Executive Committee. You will receive a response within 10 working days to advise you whether your proposal has been approved.
