# Supplementary material for: Avon Longitudinal Study of Parents and Children (ALSPAC) summary education attainment values in the offspring generation
Source: Wellcome Open Res. 2026 Apr 24;11:81. Originally published 2026 Jan 26. [Version 2] doi: 10.12688/wellcomeopenres.24082.2 (PMC12933042; doi:10.12688/wellcomeopenres.24082.2)
Supplement: Peer review history

# Reviewer report: Reviewer response for version 2

**DOI:** 10.21956/wellcomeopenres.29086.r154048

**Published:** 2026-04-27

**Authors:** Rita J Iskandar

**Affiliations:** 1 Leslie Dan Faculty of Pharmacy, University of Toronto, Toronto, Canada

Thank you to the authors for the revisions.

 The manuscript has improved, particularly with the added data limitations section and further clarification on several methodological details. Overall, I find that the authors have mostly addressed the main concerns raised in the previous review, and the data note is now clearer and more useful for researchers. 

 I have a few remaining minor comments that could further improve clarity for end users:

1. **Representativeness and coverage: **The authors state that the data are "for state-maintained schools and colleges only." My understanding is that while NPD data submission is required for state-maintained schools, some pupils in private schools or other educational settings may also be included where data are submitted voluntarily. It would therefore be helpful to clarify whether the linked data are exclusively from state-maintained schools or whether they predominantly reflect state-maintained schooling but may include some participants from other settings.
2. **Timeline and data source coverage: **The authors indicate that the summary educational data cover the academic years from 1995/96 and later note that the NPD extract provided to ALSPAC covered years 1997/98 to 2011/12. This leaves unclear how users should describe the source coverage. A brief clarification would help readers understand whether educational data should be considered available from 1995/96 overall or from 1997/98 for NPD-linked data specifically.
3. **Table 4 exclusion category definitions: **Table 4 is central for users, but the distinction between “Not in dataset” and “No data” remains unclear. It would be very helpful to define these categories in a footnote, for example, whether “Not in dataset” refers to participants not linked or not present in the relevant source dataset and whether “No data” refers to linked participants without a valid score.

These are minor points and do not detract from the overall usefulness of the resource.

Are sufficient details of methods and materials provided to allow replication by others?

No

Is the rationale for creating the dataset(s) clearly described?

Partly

Are the datasets clearly presented in a useable and accessible format?

Partly

Are the protocols appropriate and is the work technically sound?

Partly

Reviewer Expertise:

epidemiology, statistical methodology, longitudinal studies, data linkage and administrative datasets, ALSPAC, social determinants of health, inflammatory skin disease, pharmaco-epidemiology, real-world evidence

I confirm that I have read this submission and believe that I have an appropriate level of expertise to confirm that it is of an acceptable scientific standard.

# Reviewer report: Reviewer response for version 2

**DOI:** 10.21956/wellcomeopenres.29086.r154049

**Published:** 2026-04-27

**Authors:** Lloyd Balbuena

**Affiliations:** 1 University of Saskatchewan, Saskatoon, Canada

All my concerns have been addressed.

Are sufficient details of methods and materials provided to allow replication by others?

Yes

Is the rationale for creating the dataset(s) clearly described?

Yes

Are the datasets clearly presented in a useable and accessible format?

Yes

Are the protocols appropriate and is the work technically sound?

Yes

Reviewer Expertise:

Mental health

I confirm that I have read this submission and believe that I have an appropriate level of expertise to confirm that it is of an acceptable scientific standard.

# Reviewer report: Reviewer response for version 1

**DOI:** 10.21956/wellcomeopenres.26569.r147125

**Published:** 2026-02-24

**Authors:** Lloyd Balbuena

**Affiliations:** 1 University of Saskatchewan, Saskatoon, Canada

This article describes the linkage of educational records to the ALSPAC study. These educational variables consist of tests and certifications obtained from school entry up to about age 18. These variables are intended as covariates instead of outcome variables themselves.

 Overall, the article is written with exceptional clarity. I particularly appreciate Table 1 that maps children's age with the UK's Key Stages. This is very helpful for researchers outside the UK. The process of obtaining the cohort members' consent, including coordinating with educational authorities is clearly described.  

 The number of cohort members who consented at age 18 to link their educational data is very impressive. All told, more than 9,000 individuals were matched up to Key Stage 5.  

 Given this high degree of completeness and the Department of Education's assessment that these educational data have low identifiability risk, I am curious why the educational variables are comparable "within the cohort only."  Having used ALSPAC data, I am aware of variables with greater sensivitity (e.g. contact with the police) are available to researchers.  If the educational variables had more granularity, these could potentially be used as outcome variables themselves instead of covariates.

Are sufficient details of methods and materials provided to allow replication by others?

Yes

Is the rationale for creating the dataset(s) clearly described?

Yes

Are the datasets clearly presented in a useable and accessible format?

Yes

Are the protocols appropriate and is the work technically sound?

Yes

Reviewer Expertise:

Mental health

I confirm that I have read this submission and believe that I have an appropriate level of expertise to confirm that it is of an acceptable scientific standard.

# Reviewer report: Reviewer response for version 1

**DOI:** 10.21956/wellcomeopenres.26569.r147126

**Published:** 2026-02-18

**Authors:** Rita J Iskandar

**Affiliations:** 1 Leslie Dan Faculty of Pharmacy, University of Toronto, Toronto, Canada

This data note reports on the derivation of summary educational performance variables using linked education records in the Avon Longitudinal Study of Parents and Children (ALSPAC). The authors introduce rank-based categorizations of educational performance across the key stages (KS) and the Early Years, intended to provide harmonized measures for use as covariates in subsequent ALSPAC analyses. The manuscript aims to describe these derived variables and their potential application in research projects using the ALSPAC data.

 The primary concerns are about (i) the reproducibility of the derived measures, (ii) justification/utility of deciles for individual-level analyses, and (iii) correct time-period anchoring. Please note that section/paragraph references refer to PDF version 1 of the data note.

**A. Major comments**

**1**. Reproducibility and risk of misuse of the derived variables

1. Please consider clarifying what the constructed variables represent: rank-based educational performance would be more appropriate than educational attainment level for the derived summary measures. While attainment measures how far one goes in education (e.g., secondary education or KS4), performance relies on scores to measure how well one performs at a given attainment level.
2. To help mitigate misinterpretation, please consider providing at least one concrete interpretation example (e.g., what it means to be in KS1 decile X vs. decile Y, including the underlying distribution) as guidance to researchers on how to interpret the data in practical terms. Also, please consider adding brief guidance cautioning against default linear treatment of deciles in regression models.
3. These summary variables capture a single performance ranking dimension; please briefly acknowledge what is (and is not) captured by this construct with implications for use.
4. For each key stage, please specify the exact underlying original score variable(s) (name and label) used to generate the summary categories.
5. Please describe the methodology explicitly: how you derived each variable and how you created the categories (e.g., within examination year vs. pooled). Please clarify why deciles do not contain approximately 10% of observations each (e.g., due to ties, missingness, or year-specific standardization) and specify the denominator and the handling of missingness and tied scores.
6. Please distinguish clearly between derivations performed by the Department for Education (DfE) to define the original score and those introduced by the authors (e.g., proration or standardization steps).
7. Please state explicitly how these variables are intended to be used analytically (e.g., covariate only), and note that analyses requiring detailed educational definitions (exposure or outcome, for example) should use the original underlying measures to reduce the risk that users rely on the summaries when the underlying measures are required.

**2**. Deciles rationale

1. While it may be common to use deciles for school-level rankings (system-level reporting), individual-level epidemiological analyses typically rely on continuous scores (e.g., GPA) or broader categories (e.g., tertiles/quartiles/quintiles) for interpretability, minimizing data sparsity, and model stability. Please justify your choice.
2. Please consider offering quartiles/quintiles and/or a continuous standardized measure to improve interpretability and analytic robustness.
3. Please comment on potential implications of deciles for small-cell issues, overparameterization, and inconsistent downstream merging of categories across studies.

**3**. Time-period anchoring for ALSPAC’s offspring generation (G1)

1. The education system narrative would benefit from clearer anchoring to the ALSPAC G1 era. Please ensure descriptions reflect what applied during G1 schooling.
2. Please clarify statements regarding compulsory education or training (e.g., KS5 policies seem contradictory between the introduction section, paragraph 3, and the next section, ALSPAC recruitment and consent, paragraph 3) and whether these applied to G1. This is important for users to understand which KS summary variable reflects performance on the final mandatory national examinations.

**4**. Potential inconsistencies

1. The manuscript states that KS4 data are available since 2010, whereas G1 GCSE timing appears earlier (approximately 2006–2009). Please clarify what “available since 2010” refers to and confirm whether G1 KS4 performance scores are fully captured. In a similar vein, please confirm the applied coverage to other KS datasets.
2. Please clarify and harmonize the timeline of the English educational system: you note education starts at Year 1 (age 5 years) but also describe Early Years provision from age 3 years. A brief distinction between Early Years provision and compulsory schooling (from KS1/Year 1) would prevent confusion for non-UK readers.
3. Please clarify the educational level of KS5 (further education vs. secondary education: Summary variables KS5 subsection vs. Introduction, paragraph 3, respectively). Please also clarify what "entries" represent in this context.
4. Please consider framing the KS4 educational performance as the standard nomenclature of “GCSE or equivalent.” The emphasis on GNVQ (a retired legacy qualification) may confuse readers unless its relevance is explained briefly.

**5**. Tables

1. Table 1: please clarify structure and labeling (e.g., “testing procedures,” some vs. most in the same cell, add “(years)” after age), consider adding education level, and include the exact examination score used to derive summary categories at each KS.
2. Table 2: please consider replacing “matched participants” with “participants with linked educational data,” and clarify that Ns reflect the same cohort progressing across stages (not unique children).
3. Table 4: please replace “0%” with “<1%” where n>0, define abbreviations (e.g., “Trip/quad”), and replace internal variable codes with readable labels plus a mapping.
4. Overall, please ensure tables allow readers to understand what was measured and how the derived variables were constructed.

**6**. Representativeness

1. Because inclusion in the derived variables depends on successful linkage and availability of valid scores, it would be helpful to describe explicitly who is represented and who is not (e.g., voluntary opt-in for private schools, exclusion of home-schooled children and of those with linked but missing scores). This would support appropriate interpretation and help users consider potential selection mechanisms in downstream analyses.
2. Please consider distinguishing between “not linked (instead of not in dataset)” and “linked but invalid/missing score,” and consider adding categories reflecting these differences where feasible.

**B. Minor comments**

1. A few minor revisions would improve clarity and consistency: please consider “mandatory” rather than “compulsory,” define “expected attainment,” and use terminology consistently throughout. Please also clarify the proration expression and expand abbreviations at first mention (e.g., TRE). Finally, modest presentation refinements (title/abstract to better reflect the focus on the derived variables, reducing repetition, maintaining a consistent focus on the English education system, and small table clean-up, such as adding aligned footnotes) would improve readability for end users.

While the creation of summary educational performance variables has the potential to be a useful resource for researchers working with ALSPAC data, the data note in its current form does not yet provide sufficient methodological detail or justification to support meaningful or reliable analytic use. The derivation of the decile-based measures is insufficiently specified. In addition, the choice of deciles is not well motivated and may be overly granular for typical epidemiologic applications, limiting interpretability and raising small-cell and overfitting concerns. Usability as covariates would improve with broader categories (e.g., quartiles or quintiles) or a continuous standardized measure. Substantial revisions are therefore needed to fully specify derivation steps and refine the categorization to enhance transparency, interpretability, and analytic value.

 I therefore recommend approval with reservations, contingent on major clarification and restructuring of the derived variables and their documentation.

Are sufficient details of methods and materials provided to allow replication by others?

No

Is the rationale for creating the dataset(s) clearly described?

Partly

Are the datasets clearly presented in a useable and accessible format?

Partly

Are the protocols appropriate and is the work technically sound?

Partly

Reviewer Expertise:

epidemiology, statistical methodology, longitudinal studies, data linkage and administrative datasets, ALSPAC, social determinants of health, inflammatory skin disease, pharmaco-epidemiology, real-world evidence

I confirm that I have read this submission and believe that I have an appropriate level of expertise to confirm that it is of an acceptable scientific standard, however I have significant reservations, as outlined above.

# Reviewer report: Reviewer response for version 1

**DOI:** 10.21956/wellcomeopenres.26569.r145773

**Published:** 2026-02-03

**Authors:** Xanthe Hunt

**Affiliations:** 1 Africa Health Research Institute (AHRI), Umbilo, South Africa

This data note is clearly written, and of high potential utility to other researchers seeking to include educational attainment variables in the context of longitudinal linked datasets.

 A few issues need to be attended to:

1. The paper states ‘Note that these consent figures represent a point in time; consent is ongoing, and numbers have changed over time. The available data set is only of those who explicitly consented and those who chose not to opt-out.’ How frequently is the available dataset updated to reflect changes in context?
2. The study notes that data for educational attainment were taken from the NPD - a longitudinal database of pupils who attend state-maintained schools and colleges - what of pupils in non-state schools?
3. It would be useful if the authors could explain how ‘bad matches’ could arise, and exactly how many there were (beyond ‘a small number’).
4. It would also be helpful if the authors could explain what accounts for the differences by KS in the number linked? If this has to do with the following (Participants might be excluded from a KS, i.e. not have a valid score, if they were not matched in that KS dataset, or were absent, ineligible, working below the level of the test, unable to access the test, had left, or their data was missing or null in the DfE dataset), then is it possible to tabulate the reasons for exclusion for each missing participant? Data on pupils working below the level of the test, for instance, might be very useful in the context of analyses exploring barriers top learning, or disability.
5. An explanation for why teaching assessments and spelling tests were not included is needed.
6. For a non-UK reader, it is helpful to elaborate on why the total possible scores differ by subject (English max 100 + Maths max 100 + Science max 80)?
7. This short paragraph could be rewritten for clarity - the sudden introduction of quotations is confusing without any modifiers: “The capped scores equate to 8 A* GCSEs (8 A* = 58 x 8 = 464), but “In practice 0.05% of the sample have scores slightly above 464 which may result from students taking qualifications which are more challenging than GCSEs (e.g. AS levels), or from small data errors.””

Are sufficient details of methods and materials provided to allow replication by others?

Yes

Is the rationale for creating the dataset(s) clearly described?

Yes

Are the datasets clearly presented in a useable and accessible format?

Yes

Are the protocols appropriate and is the work technically sound?

Yes

Reviewer Expertise:

adolescent mental health; global health; digital health; measurement in mental health; disability

I confirm that I have read this submission and believe that I have an appropriate level of expertise to confirm that it is of an acceptable scientific standard, however I have significant reservations, as outlined above.
